# Late Recurrence of Breast Cancer Nearly 30 Years After Surgery: Mediastinal and Hilar Lymph Node Metastases Diagnosed Using Endobronchial Ultrasound-Guided Transbronchial Needle Aspiration

**DOI:** 10.7759/cureus.97308

**Published:** 2025-11-20

**Authors:** Tomoyuki Araya, Toshiyuki Kita, Takayuki Higashi, Ryo Hara, Tsukasa Ueda, Hazuki Takato

**Affiliations:** 1 Respiratory Medicine, National Hospital Organization Kanazawa Medical Center, Kanazawa, JPN

**Keywords:** ebus-tbna (endobronchial ultrasound-transbronchial needle aspiration), hilar lymphadenopathy, late recurrence, mediastinal lymph node metastasis, metastatic breast cancer

## Abstract

Late recurrence of breast cancer occurring more than two decades after curative surgery is rare, but in the era of prolonged survival, clinicians may increasingly encounter such cases. Mediastinal or hilar recurrence may radiologically mimic primary lung cancer; however, histological confirmation using endobronchial ultrasound-guided transbronchial needle aspiration (EBUS-TBNA) enables accurate diagnosis with minimal invasiveness. We report the cases of two patients who developed mediastinal and hilar lymph node metastases nearly 30 years after breast cancer surgery, for whom EBUS-TBNA played a crucial diagnostic role. A 68-year-old woman with a history of right mastectomy for stage I (T1aN0M0) breast cancer 30 years earlier presented with cough and hemoptysis. Chest computed tomography revealed multiple pulmonary nodules, right hilar lymphadenopathy, and sclerotic bone lesions. Fluorodeoxyglucose positron emission tomography/computed tomography showed intense uptake in the right hilar lymph nodes and bones, whereas pulmonary nodules demonstrated only mild uptake. EBUS-TBNA of station 12R revealed adenocarcinoma positive for estrogen and progesterone receptors and negative for human epidermal growth factor receptor 2, confirming metastatic breast carcinoma. The patient was treated with abemaciclib plus fulvestrant, achieving durable disease control for 2.5 years. A 70-year-old woman who had undergone left mastectomy 27 years earlier presented with a dry cough. EBUS-TBNA of stations 4R and 7 confirmed metastatic breast carcinoma. She received nab-paclitaxel followed by trastuzumab deruxtecan, achieving disease control for 20 months. These cases demonstrate that even very late recurrences nearly three decades after surgery can achieve prolonged survival when appropriately diagnosed and optimally treated. EBUS-TBNA is a valuable and minimally invasive diagnostic tool for confirming such a late recurrence.

## Introduction

Breast cancer is one of the most prevalent malignancies worldwide, and advances in early detection and systemic therapy have significantly improved long-term survival [1]. As a result, an increasing number of patients remain at risk for late recurrence, defined as recurrence occurring more than 10 years after initial curative treatment [2]. Among these, hormone receptor-positive tumors are particularly prone to relapse even beyond two decades, reflecting prolonged micrometastatic dormancy [3,4].

Late recurrences most commonly involve the bone, lung, pleura, and liver, whereas mediastinal and hilar lymph node (LN) metastases are relatively uncommon [5-7]. In a large metastatic-pattern analysis of adenocarcinomas by Hess et al., mediastinal LN metastases were observed in only 5.7% of breast cancer cases, underscoring their rarity [6]. Consistent with this, Yamashita et al. documented mediastinal involvement mimicking a primary mediastinal tumor, highlighting the diagnostic pitfall in breast cancer survivors [7]. During post-curative surveillance, Kim et al. showed that newly detected isolated mediastinal LN enlargements are malignant in roughly one-third of cases and identified clinical-imaging factors (e.g., high SUV on PET, hormone receptor negativity) that aid triage for tissue diagnosis [8]. Complementing these data, Argento et al. reported a small retrospective series of 64 breast cancer patients undergoing endobronchial ultrasound-guided transbronchial needle aspiration (EBUS-TBNA) for mediastinal lymphadenopathy, with metastatic disease confirmed in approximately half and with frequent receptor discordance between primary and thoracic metastases [9]. Collectively, although lung parenchymal metastases are relatively frequent, coexisting mediastinal/hilar LN involvement remains uncommon and can be radiologically difficult to distinguish from primary lung cancer.

Because such thoracic lesions may mimic primary lung cancer, histopathological confirmation is essential. EBUS-TBNA has emerged as a minimally invasive, high-yield modality for unexplained mediastinal lymphadenopathy [10,11], and its utility in confirming metastatic breast carcinoma has been increasingly recognized in contemporary series [9].

Here, we present the cases of two patients who developed mediastinal and hilar LN metastases accompanied by pulmonary lesions nearly three decades after breast cancer surgery, emphasizing the need to consider very late recurrence and the diagnostic value of EBUS-TBNA in this setting.

The case series was approved by the ethics committee of the NHO Kanazawa Medical Center. Written informed consent was obtained from both patients for publication of this report and any accompanying images. The study was conducted ethically in accordance with the World Medical Association Declaration of Helsinki.

## Case presentation

Case 1

A 68-year-old woman presented with cough and hemoptysis. She had undergone right mastectomy for breast cancer 30 years earlier, diagnosed as stage I (T1aN0M0). The staging of breast cancer was determined according to the tumor-node-metastasis (TNM) system described by Harris et al. [12], which corresponded to the 4th edition of the Union for International Cancer Control (UICC) TNM Classification (1992) and is freely available for academic and non-commercial use. She had received no adjuvant therapy. She had no history of smoking and no family history of malignancy. Laboratory tests revealed almost normal results, including normal levels of carcinoembryonic antigen (CEA), cytokeratin 19 fragment (CYFRA 21-1), and pro-gastrin-releasing peptide (ProGRP), except for a borderline elevation in lactate dehydrogenase (Table 1).

**Table 1:** Laboratory findings of case 1 Laboratory data on admission showing values mostly within the normal range except for a borderline elevation in LDH. WBC, white blood cell; RBC, red blood cell; Hb, hemoglobin; Ht, hematocrit; Plt, platelet; CRP, C-reactive protein; T-Bil, total bilirubin; TP, total protein; ALP, alkaline phosphatase; AST, aspartate aminotransferase; ALT, alanine aminotransferase; LDH, lactate dehydrogenase; Alb, albumin; Na, sodium; K, potassium; Cl, chloride; BUN, blood urea nitrogen; Cr, creatinine; eGFR, estimated glomerular filtration rate; UA, uric acid; CEA, carcinoembryonic antigen; CYFRA 21-1, cytokeratin 19 fragment; ProGRP, pro-gastrin-releasing peptide

Parameter	Measured Value	Reference Range
WBC	8,800/µL	4,500–9,000/µL
Neutrophil	72.9%	38–74%
Lymphocyte	21.7%	16.5–49.5%
Monocyte	4.1%	5–10%
Eosinophil	0.7%	0–10%
Basophil	0.6%	0–2%
RBC	4.26 ×10⁶/µL	3.82–5.00 ×10⁶/µL
Hb	13.6 g/dL	11.7–14.6 g/dL
Ht	41.3%	34.3–44.2%
Plt	24.7 ×10⁴/µL	15–35 ×10⁴/µL
CRP	0.06 mg/dL	0–0.4 mg/dL
T-Bil	0.9 mg/dL	0.3–1.2 mg/dL
TP	7.1 g/dL	6.7–8.3 g/dL
ALP	85 U/L	38–113 U/L
AST	17 U/L	13–33 U/L
ALT	15 U/L	6–27 U/L
LDH	230 U/L	119–229 U/L
Alb	4.2 g/dL	4.0–5.0 g/dL
Na	139 mEq/L	135–149 mEq/L
K	3.8 mEq/L	3.5–4.9 mEq/L
Cl	99 mEq/L	96–108 mEq/L
BUN	17.6 mg/dL	8–22 mg/dL
Cr	0.56 mg/dL	0.5–0.8 mg/dL
eGFR	80.5 mL/min/L	60–100 mL/min/L
UA	4.2 mg/dL	2.3–7.0 mg/dL
D-dimer	0.9 µg/mL	0–1 µg/mL
CEA	1.8 ng/mL	<3.5 ng/mL
CYFRA 21-1	2.0 ng/mL	<3.5 ng/mL
ProGRP	32.6 pg/mL	<80 pg/mL

**Figure 1:**
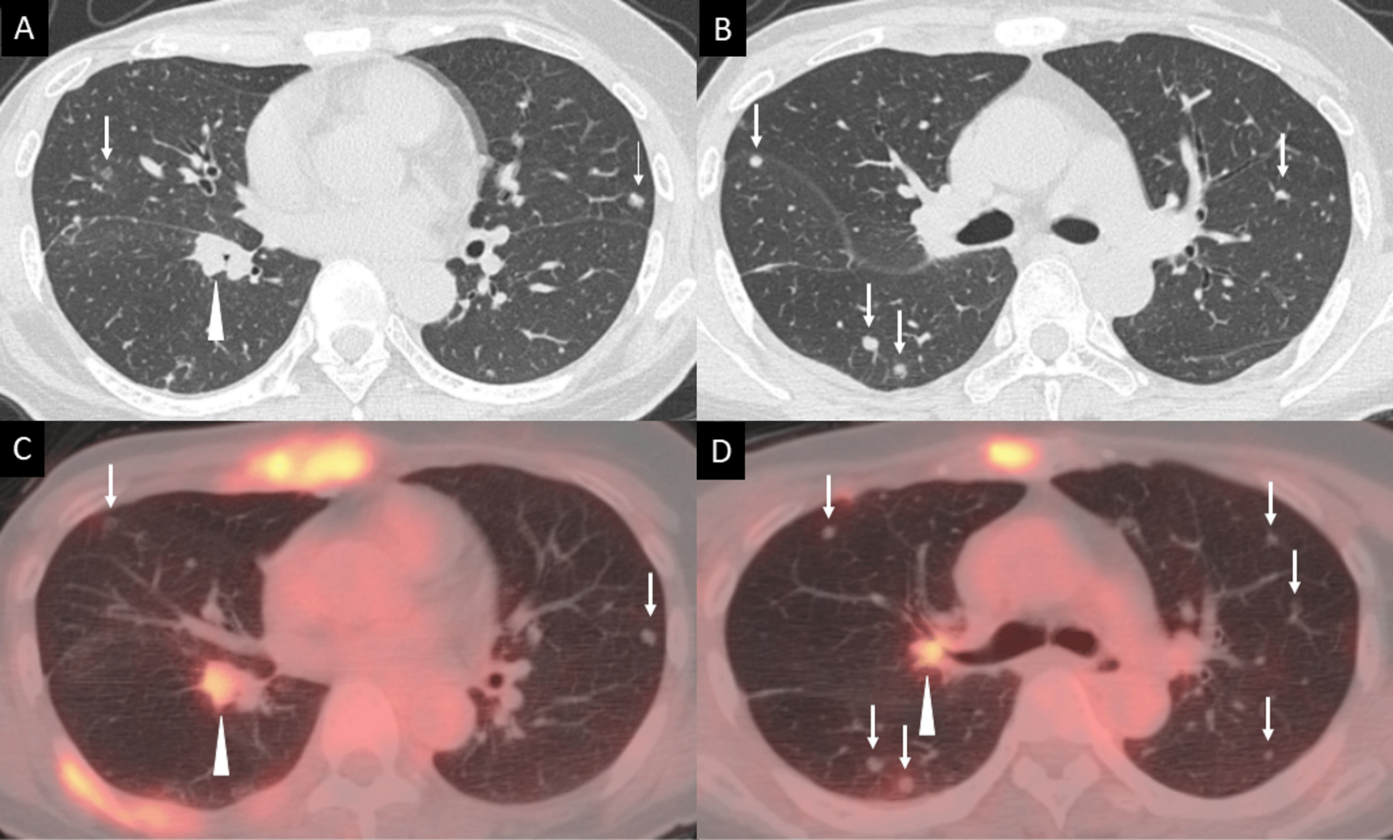
Chest CT and PET/CT findings of case 1 (A, B) Chest CT images showing multiple small pulmonary nodules measuring 2–5 mm in diameter (arrows) and right hilar lymphadenopathy (arrowheads). The right hilar lymph node (station 12R) is indicated in panel A. (C, D) PET/CT images demonstrating intense FDG uptake in the hilar lymph nodes (arrowheads) and sclerotic bone metastases, whereas the pulmonary nodules exhibit only mild uptake (arrows). CT, computed tomography; PET/CT, positron emission tomography/computed tomography; FDG, fluorodeoxyglucose

Chest computed tomography (CT) demonstrated multiple pulmonary nodules (measuring 2-5 mm in diameter), right hilar lymphadenopathy, and sclerotic bone lesions (Figures 1A, 1B). Fluorodeoxyglucose positron emission tomography/computed tomography (FDG-PET/CT) revealed intense FDG uptake in the right hilar LNs and bones, whereas the pulmonary nodules showed only mild uptake (Figures 1C, 1D).

**Figure 2:**
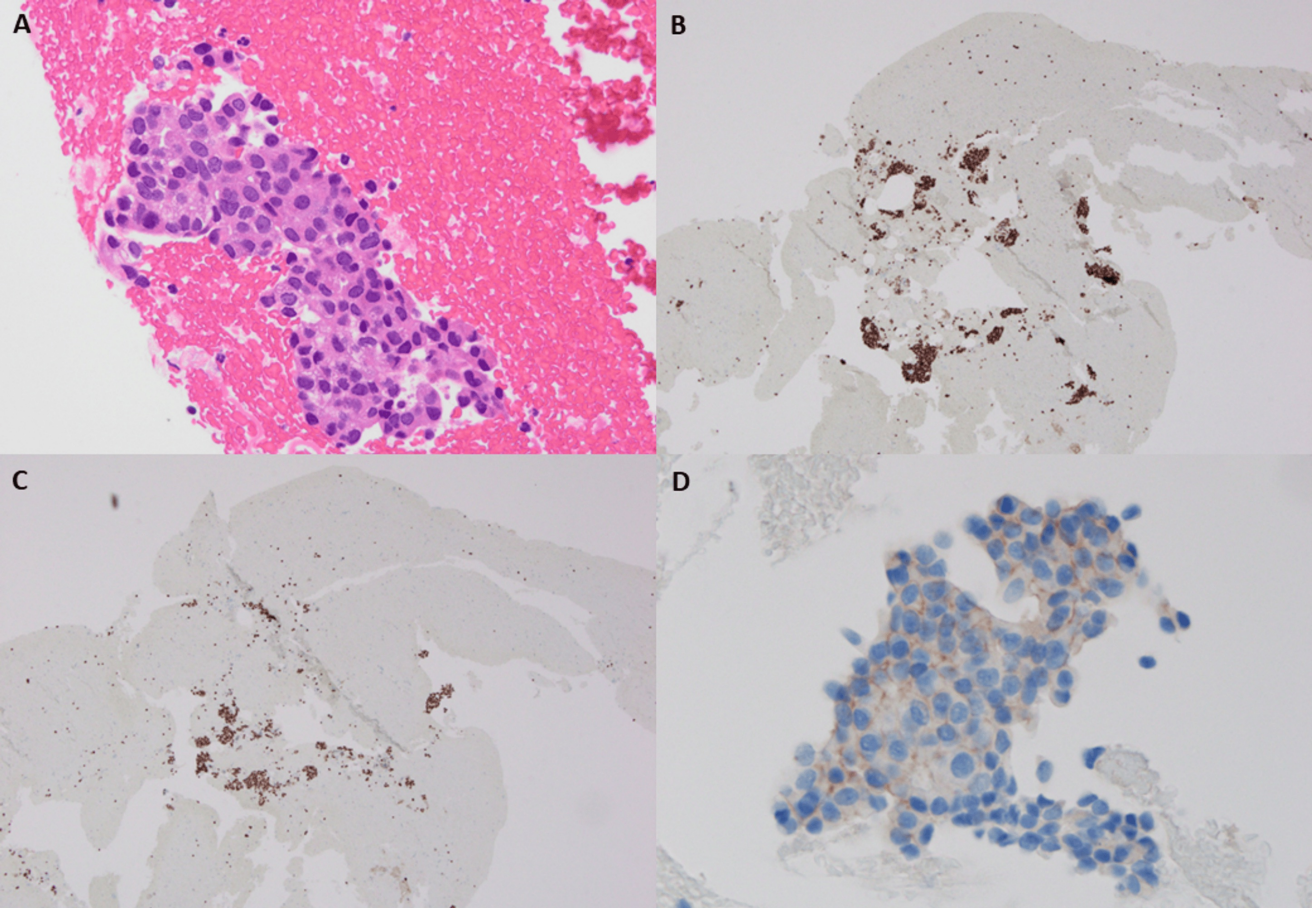
Histopathological and immunohistochemical findings of case 1 (A) Hematoxylin and eosin (H&E)–stained specimen obtained by EBUS-TBNA of the 12R lymph node, showing clusters of adenocarcinoma cells with glandular and trabecular architecture (original magnification ×400). (B) IHC for ER demonstrates diffuse nuclear positivity in tumor cells (×40). (C) PR staining is also positive in tumor nuclei (×40). ER and PR expressions were evaluated by immunohistochemistry in accordance with the ASCO/CAP guidelines for hormone receptor testing in breast cancer [13]. (D) HER2 immunostaining shows a weak, incomplete membranous pattern corresponding to score 1+ (×400). HER2 immunostaining performed with a VENTANA anti-HER2/neu (4B5) antibody shows a weak, incomplete membranous pattern corresponding to score 1+, interpreted according to the ASCO/CAP guidelines [14]. EBUS-TBNA, endobronchial ultrasound-guided transbronchial needle aspiration; IHC, immunohistochemistry; ER, estrogen receptor; PR, progesterone receptor; HER2, human epidermal growth factor receptor 2; ASCO/CAP, American Society of Clinical Oncology/College of American Pathologists

No abnormal uptake was observed in the abdominal or pelvic organs. During bronchoscopy, no active endobronchial bleeding was observed. However, marked mucosal edema and capillary dilatation were noted in the bronchial epithelium of the right lower lobe, likely secondary to compression and inflammation associated with the metastatic station 12R LN. These vascular changes were considered the most plausible source of hemoptysis. Based on these findings, primary lung cancer in the right lower lobe with multiple pulmonary and bone metastases was clinically suspected. However, EBUS-TBNA of station 12R revealed clusters of adenocarcinoma cells with glandular and trabecular architecture. Immunohistochemistry showed positivity for estrogen receptor (ER) and progesterone receptor (PR) and negativity for human epidermal growth factor receptor 2 (HER2; score 1+), thyroid transcription factor-1 (TTF-1), napsin A, p40, and neural cell adhesion molecule (CD56), confirming metastatic breast carcinoma (Figure 2). ER, PR, and HER2 expressions were interpreted according to the American Society of Clinical Oncology/College of American Pathologists (ASCO/CAP) guidelines for hormone receptor and HER2 testing in breast cancer [13,14].

Following the diagnosis, the patient was started on abemaciclib plus fulvestrant therapy, achieving durable disease control for 2.5 years to date.

Case 2

A 70-year-old woman, who had undergone left mastectomy for stage I (T1cN0M0) breast cancer followed by adjuvant hormonal therapy for four years 27 years earlier, presented with progressive dry cough. The staging of breast cancer was determined according to the TNM system described by Harris et al. [12], which corresponded to the 4th edition of the UICC TNM Classification (1992) and is freely available for academic and non-commercial use. She had no history of smoking and no family history of malignancy. Laboratory tests showed a mildly elevated C-reactive protein level, a markedly increased D-dimer level (18.1 µg/mL), and a high CYFRA 21-1 concentration (11.0 ng/mL), while CEA and ProGRP levels were within normal limits, findings consistent with the initial clinical suspicion of primary lung cancer (Table 2).

**Table 2:** Laboratory findings of case 2 Laboratory data on admission showing mild anemia, mildly elevated CRP, and marked elevations in D-dimer and CYFRA 21-1, with other parameters mostly within normal limits. WBC, white blood cell; RBC, red blood cell; Hb, hemoglobin; Ht, hematocrit; Plt, platelet; CRP, C-reactive protein; T-Bil, total bilirubin; TP, total protein; ALP, alkaline phosphatase; AST, aspartate aminotransferase; ALT, alanine aminotransferase; LDH, lactate dehydrogenase; Alb, albumin; Na, sodium; K, potassium; Cl, chloride; BUN, blood urea nitrogen; Cr, creatinine; eGFR, estimated glomerular filtration rate; UA, uric acid; CEA, carcinoembryonic antigen; CYFRA 21-1, cytokeratin 19 fragment; ProGRP, pro-gastrin-releasing peptide

Parameter	Measured Value	Reference Range
WBC	5,700/µL	4,500–9,000/µL
Neutrophil	58.8%	38–74%
Lymphocyte	24.9%	16.5–49.5%
Monocyte	11.6%	5–10%
Eosinophil	4.0%	0–10%
Basophil	0.7%	0–2%
RBC	3.71 ×10⁶/µL	3.82–5.00 ×10⁶/µL
Hb	10.9 g/dL	11.7–14.6 g/dL
Ht	34.1%	34.3–44.2%
Plt	26.5 ×10⁴/µL	15–35 ×10⁴/µL
CRP	0.74 mg/dL	0–0.4 mg/dL
T-Bil	0.8 mg/dL	0.3–1.2 mg/dL
TP	6.9 g/dL	6.7–8.3 g/dL
ALP	75 U/L	38–113 U/L
AST	19 U/L	13–33 U/L
ALT	10 U/L	6–27 U/L
LDH	189 U/L	119–229 U/L
Alb	4.0 g/dL	4.0–5.0 g/dL
Na	138 mEq/L	135–149 mEq/L
K	4.4 mEq/L	3.5–4.9 mEq/L
Cl	101 mEq/L	96–108 mEq/L
BUN	12.3 mg/dL	8–22 mg/dL
Cr	0.69 mg/dL	0.5–0.8 mg/dL
eGFR	50.7 mL/min/L	60–100 mL/min/L
UA	3.8 mg/dL	2.3–7.0 mg/dL
D-dimer	18.1 µg/mL	0–1 µg/mL
CEA	3.0 ng/mL	<3.5 ng/mL
CYFRA 21-1	11.0 ng/mL	<3.5 ng/mL
ProGRP	40.2 pg/mL	<81 pg/mL

**Figure 3:**
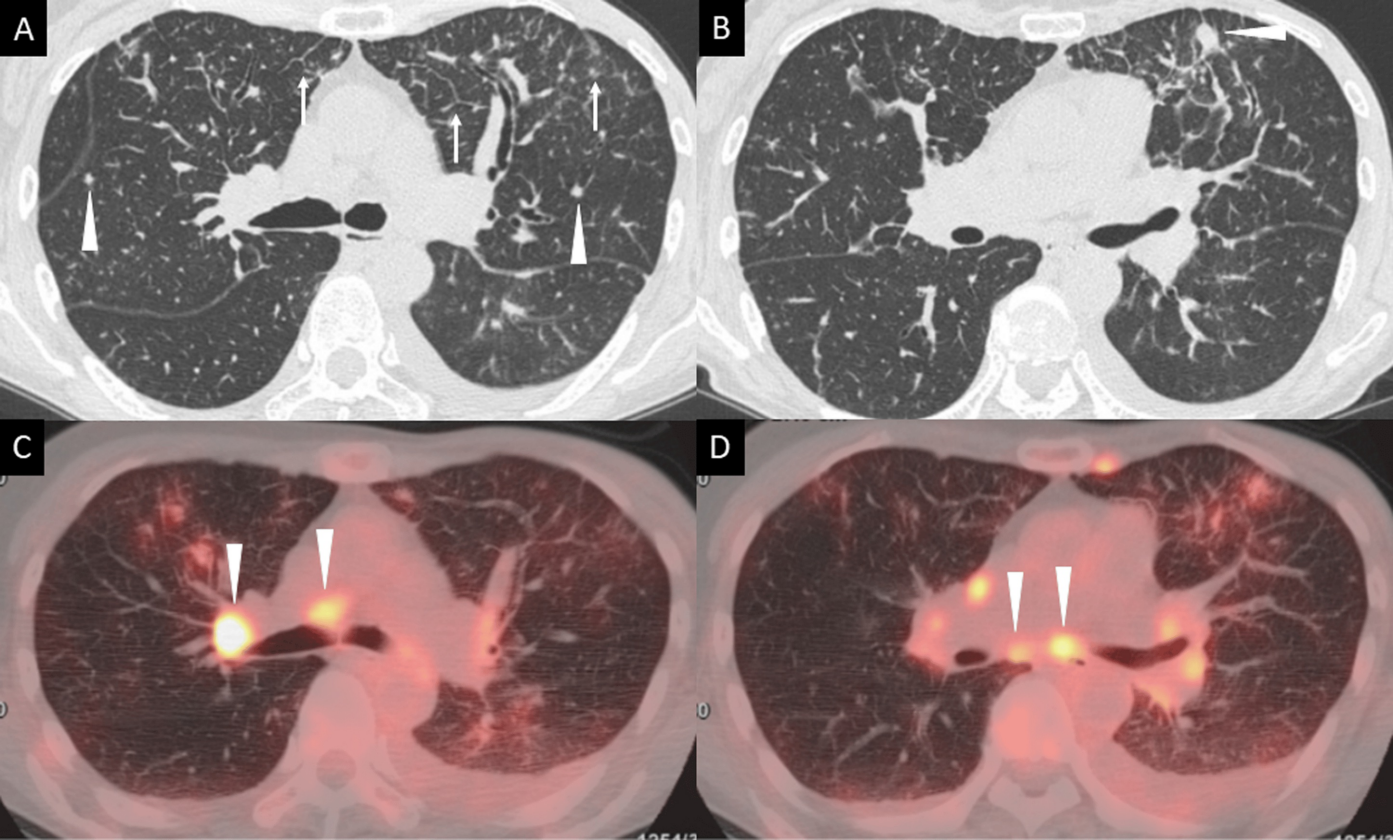
Chest CT and PET/CT findings of case 2 (A) Chest CT image showing multiple pulmonary nodules measuring 3–9 mm in diameter (arrowheads) and thickening of the interlobular septa with fine granular opacities (arrows), findings suggestive of carcinomatous lymphangitis. (B) CT image demonstrating a representative pulmonary nodule (arrowhead) corresponding to the largest lesion (9 mm) in the right lung, with additional smaller nodules present bilaterally. (C, D) PET/CT images showing enlargement and abnormal FDG uptake in multiple LNs, including stations 4R and 7 (arrowheads). Strong FDG accumulation is observed in the LNs, whereas the pulmonary lesions show only mild uptake. CT, computed tomography; PET/CT, positron emission tomography/computed tomography; FDG, fluorodeoxyglucose

Chest CT revealed multiple small pulmonary nodules measuring 3-9 mm in diameter and findings suggestive of lymphangitic spread, along with mediastinal and hilar LN enlargement (Figures 3A, 3B). FDG-PET/CT demonstrated intense FDG uptake in the mediastinal and hilar LNs, as well as increased uptake in the pulmonary lesions (Figures 3C, 3D).

In addition, FDG accumulation was observed in the abdominal para-aortic LNs, although these findings are not shown.

**Figure 4:**
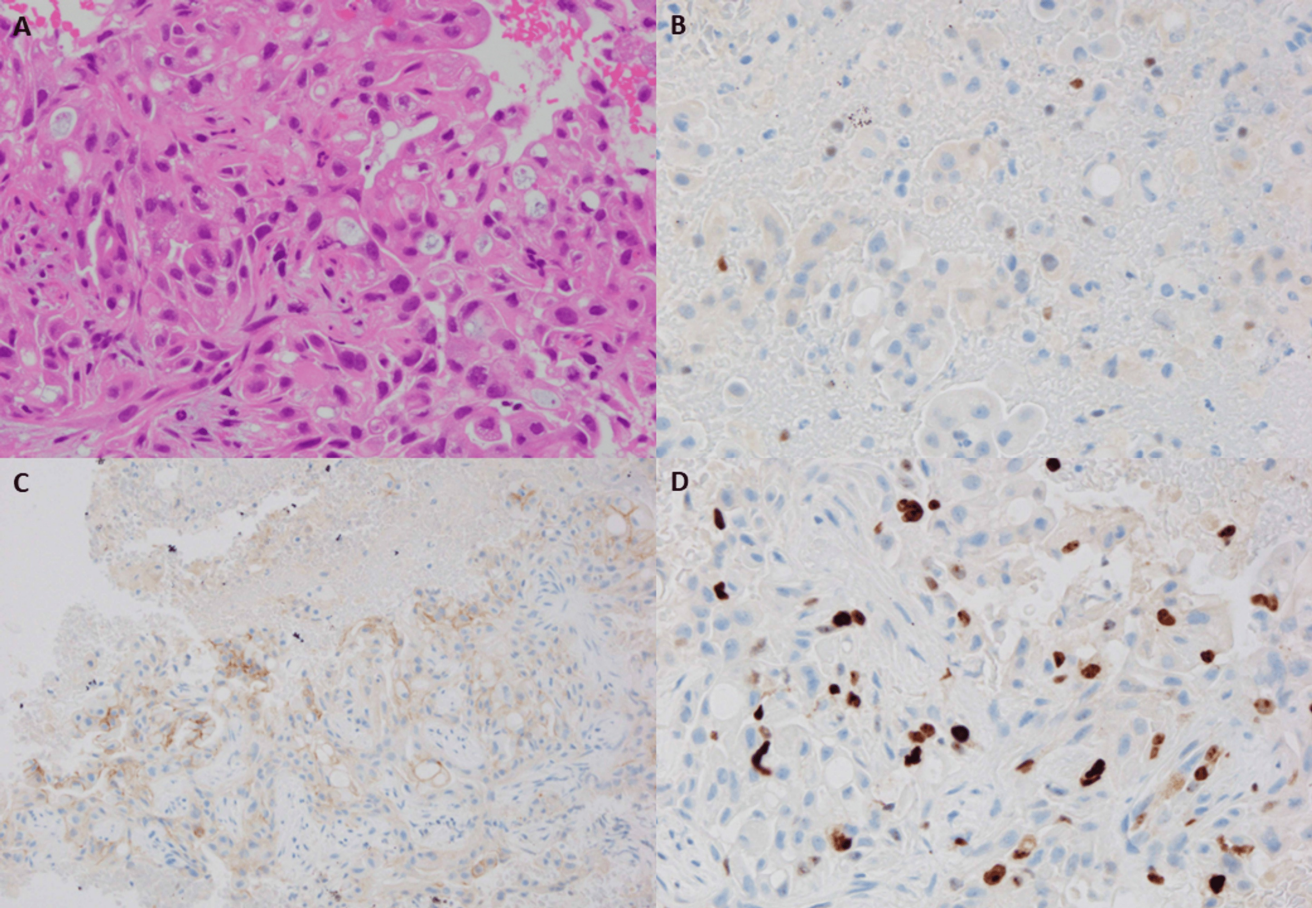
Histopathological and immunohistochemical findings of case 2 (A) Hematoxylin and eosin (H&E)-stained specimen obtained from station 4R by EBUS-TBNA showing clusters of carcinoma cells with round to irregular nuclei (×400). The sample from station 7 showed similar findings. (B–D) IHC demonstrating positivity for (GATA3 (B, ×400), HER2 IHC score 1+ (negative) (C, ×200), and a MIB-1 labeling index of approximately 30% (D, ×400). HER2 immunostaining performed with a VENTANA anti-HER2/neu (4B5) antibody shows a weak, incomplete membranous pattern corresponding to score 1+, interpreted according to the ASCO/CAP guidelines [14]. The Ki-67 labeling index was evaluated according to the recommendations of the International Ki-67 in Breast Cancer Working Group [15]. EBUS-TBNA, endobronchial ultrasound-guided transbronchial needle aspiration; IHC, immunohistochemistry; GATA3, GATA-binding protein 3; HER2, human epidermal growth factor receptor 2; ASCO/CAP, American Society of Clinical Oncology/College of American Pathologists

Based on these findings, primary lung cancer with carcinomatous lymphangitis was initially suspected. However, EBUS-TBNA of stations 4R and 7 revealed adenocarcinoma composed of cells with eosinophilic cytoplasm and irregular nuclei. The histological specimen presented in this report was obtained from station 4R, while the sample from station 7 showed similar findings. Immunohistochemistry showed positivity for GATA-binding protein 3 (GATA3) and negativity for ER, PR, HER2 (score 1+), TTF-1, napsin A, and p40. The MIB-1 labeling index was approximately 30%. These findings confirmed metastatic breast carcinoma (Figure 4).

Because the radiological findings strongly suggested carcinomatous lymphangitis, a transbronchial cryobiopsy was performed from the right B8a segment; however, no malignant cells were identified. Additional biomarker analyses revealed negative programmed death-ligand 1 expression, assessed using the 22C3 antibody clone in accordance with the ASCO guideline update for biomarkers in metastatic breast cancer [16]. BRCA1/2 testing using the BRACAnalysis CDx assay, which has been validated as a companion diagnostic tool [17], revealed no pathogenic variants [17].

Following histopathological confirmation of metastatic breast carcinoma, the patient received nab-paclitaxel as first-line chemotherapy and trastuzumab deruxtecan as second-line therapy. The disease remained controlled for 16 months. At that time, overt pancreatic cancer developed, and systemic therapy was subsequently shifted toward pancreatic cancer treatment. She has continued therapy for four months to date, and no evidence of recurrent breast cancer has been observed. Thus, she has survived for 20 months after the diagnosis of breast cancer recurrence.

## Discussion

Breast cancer can recur even decades after curative surgery, sometimes manifesting as mediastinal or hilar lymphadenopathy with or without other organ involvement. Such recurrence patterns may radiologically resemble primary lung cancer, posing a major diagnostic challenge. In this context, EBUS-TBNA provides a safe, minimally invasive, and reliable means of obtaining tissue for histopathological and immunohistochemical confirmation.

Very late recurrence is thought to reflect tumor dormancy, in which micrometastatic cells remain quiescent for years before reactivation under hormonal or immunologic influences. In a large Danish cohort, Pedersen et al. reported that breast cancer recurrence can occur even 10-32 years after primary surgery, particularly among ER-positive patients, with approximately 16% experiencing very late relapse, underscoring the lifelong risk of recurrence in this subgroup [18]. They further demonstrated that larger tumor size, LN positivity, and ER-positive status were independent risk factors for late recurrence. In our report, case 1 (T1aN0M0, ER-positive) fits this risk profile, whereas case 2 (T1cN0M0, ER-negative) does not. The very late recurrence in case 2 is therefore unusual and highlights that, although rare, late relapse can also occur in ER-negative early-stage breast cancer.

Although bone and lung are the predominant sites of late recurrence, mediastinal and hilar LN metastases can occasionally occur and mimic lung adenocarcinoma both radiologically and cytologically. Immunohistochemical analysis is therefore critical to distinguish metastatic breast carcinoma from lung adenocarcinoma; markers such as ER, PR, and GATA3 support a breast origin, whereas TTF-1 and napsin A favor lung adenocarcinoma [9].

EBUS-TBNA has proven to be an indispensable diagnostic tool for evaluating mediastinal and hilar lymphadenopathy, providing high diagnostic accuracy comparable to mediastinoscopy but with less invasiveness and greater safety [10,11]. In our two patients, both of whom experienced recurrence nearly three decades after mastectomy, EBUS-TBNA enabled definitive diagnosis of metastatic breast carcinoma that radiologically mimicked primary lung cancer. These cases illustrate that even very late recurrences nearly three decades after surgery can achieve prolonged survival when appropriately diagnosed and optimally treated, underscoring the importance of maintaining long-term vigilance and the diagnostic value of EBUS-TBNA as a first-line modality in breast cancer survivors with new thoracic lesions.

## Conclusions

Breast cancer may recur even decades after curative surgery. When mediastinal or hilar lymphadenopathy is detected in long-term survivors, recurrence should be considered regardless of the interval since surgery. EBUS-TBNA provides minimally invasive and reliable histopathological confirmation, making it an essential first-line diagnostic tool for detecting very late thoracic recurrence of breast cancer. Furthermore, these cases illustrate that even very late recurrences nearly three decades after surgery can achieve prolonged survival when appropriately diagnosed and optimally treated. Maintaining clinical suspicion and promptly establishing a tissue diagnosis are key to enabling effective therapy and extending survival in this rare but important clinical scenario.
